# Finite Element Investigation on Cutting Force and Residual Stress in 3D Elliptical Vibration Cutting Ti6Al4V

**DOI:** 10.3390/mi13081278

**Published:** 2022-08-08

**Authors:** Shiyu Li, Jinguo Han, Haiqiang Yu, Jinhui Wang, Mingming Lu, Yebing Tian, Jieqiong Lin

**Affiliations:** 1 School of Mechanical Engineering, Shandong University of Technology, Zibo 255049, China; 2 Shandong Provincial Key Laboratory of Precision Manufacturing and Non-Traditional Machining, Shandong University of Technology, Zibo 255049, China; 3 School of Mechatronic Engineering, Changchun University of Technology, Changchun 130012, China

**Keywords:** Ti-6Al-4V alloy, 3D EVC, cutting force, residual stress, finite element method

## Abstract

Titanium alloy is a typical difficult-to-machine material with features of superhigh strength and hardness, and low elastic modulus. It is difficult to guarantee the processing quality and efficiency due to the high cutting force and tool wear in conventional cutting. Elliptical vibration cutting (EVC) as an effective method can improve the machinability of titanium alloys. In this paper, the finite element method (FEM) was adopted to study the cutting force and residual stress of 3D EVC in machining of Ti6Al4V. The Johnson-Cook constitutive model was utilized to illustrate the plastic behavior of Ti6Al4V alloy. The kinematics of the 3D EVC was described, and then the influence of various cutting speeds, vibration amplitudes, vibration frequencies and depths of cut on cutting force and residual stress were carried out and analyzed. The simulation results show that the cutting speed, vibration amplitude *a*, vibration frequency and depth of cut have larger effect on principal force. In addition, the compressive stress layer can be easily obtained near the machined surface by using 3D EVC, which is helpful to improve the working performance of workpiece.

## 1. Introduction

Titanium alloy is widely used in aerospace, shipbuilding, communication equipment, medical equipment and other fields due to its low density, high strength and stable performance under extreme conditions. However, for titanium alloy it is hard to obtain the ideal machining results by traditional machining methods. The characteristics of low thermal conductivity and high elasticity of titanium alloy produce a lot of heat during the cutting period, and the friction between the tool and the workpiece is serious, which makes the cutting force too large [1]. The high chemical reactivity of titanium alloy leads to a compound wear mechanism, which aggravates tool wear [2]. These disadvantages limit the application of titanium alloy in many fields.

Aiming to improve the machinability and increase machining efficiency of titanium alloy, many researchers have studied cutting methods of titanium alloy in recent years, including laser machining [3], electrical discharge machining (EDM) [4], electron beam machining (EBM) [5], vibration cutting [6] and so on. Among these methods, elliptical vibration cutting (EVC) technology has been considered as an effective way to improve the machinability of titanium alloys. As a new material processing method, EVC was first introduced by Shamoto and Moriwaki in 1994 [7]. The characteristics of EVC is intermittent contact and separation between the tool and the workpiece within an elliptical vibration cycle. Moreover, the reversal of friction force direction on tool rake face, which is helpful in chip removal, is another interesting phenomenon [8]. EVC has the advantages of reducing cutting force [9], improving machining surface quality [10], reducing tool wear [11,12], inhibiting burr generation [13] and reducing residual stress after machining [14] compared with conventional cutting (CC) methods. In recent years, EVC has been widely used in the processing of difficult-to-machine materials [15,16,17], especially in the fabrication of micro-nano structure surfaces, which highlight great advantages [18,19,20].

However, as the commonly used EVC only moves elliptically in the machining plane, i.e., two-dimensional EVC (2D-EVC), the tool has a low degree of freedom, which has limitations in machining complex free-form surfaces. Aiming to meet the processing requirements of complicated surfaces, Shamoto introduced three-dimensional EVC (3D-EVC) technology [21]. This method is a spatial elliptical vibration cutting technique. The higher modulation ability of 3D-EVC makes it more suitable for machining free-form surfaces [22]. Compared with 2D-EVC, the more parameters of 3D-EVC can be dynamically adjusted with the characteristics of the workpiece [23]. In addition, 3D-EVC can reduce cutting force more effectively, enhance chip removal efficiency and improve the machining surface quality of the workpiece [24,25]. At present, for 3D-EVC, researchers mainly focus on the studies of optimization of machining parameters [26], reduction of tool wear [27], preparation of micro-nano structures [28] and the procession of composite materials [29]. Research on cutting force and residual stress is rare.

Generally, cutting force and residual stress are important parameters that influence the workpiece machining process and workpiece accuracy after machining. The popular methods for cutting force and residual stress studying are the experimental method and the finite element method (FEM). Compared with the experimental method, FEM not only has the characteristics of low cost and high efficiency, but also is more flexible. Therefore, FEM is favored by many researchers. Currently, there are many studies about studying the cutting mechanism of titanium alloy during machining by FEM [30,31,32,33], while few studies have been carried out on the cutting force and residual stress in 2D-EVC of titanium alloy. Xie et al. [34] used FEM to study the changes of cutting force in EVC titanium alloy with vibration frequency, amplitude and cutting speed. The results show that the tangential force decreases with the increase of vibration frequency, tangential amplitude and thrust amplitude, but decreases with the decrease of cutting speed. The positive and negative thrust force decreases with the increase of frequency and tangential amplitude but decreases with the decrease of cutting speed and thrust amplitude. Wang et al. [35] discussed the material removal mechanism of ultrasonic EVC under different cutting paths. Through simulation and experiment, the material removal mechanism of medical β titanium alloy in ultrasonic elliptical vibration cutting was studied from the aspects of cutting deformation, cutting force and residual stress. The cutting force and residual stress of EVC on some other difficult-to-machine materials are also investigated by FEM. Zhou et al. [36] established a finite element model of elliptical vibration cutting SiCp/Al composite material to study the material removal mechanism, cutting force changes and surface roughness in EVC process. The results show that EVC not only reduces the average cutting force, but also avoids the scratches caused by SiC particles on the machined surface, thus improving the machined surface quality. Kurniawan et al. [37] used FEM to study the difference between ultrasonic EVC and CC of AISI 1045. The cutting force and residual stress have been studied. The results show that the average cutting force of ultrasonic EVC increases with the increase of speed ratio and feed speed, and the average cutting force decreases with the increase of vibration amplitude. In addition, the surface residual stress of the two cutting techniques is tensile stress, and the maximum compressive stress of ultrasonic EVC process is larger than that of CC process due to the compression effect of the vibrated cutting tool.

Above all, the research on the cutting force and residual stress in 3D-EVC needs to be further studied. Therefore, FEM was adopted to investigate the effect on cutting force and residual stress of different processing parameters, such as cutting speed, vibration amplitude, vibration frequency and depth of cut.

## 2. Kinematic of 3D-EVC

**Figure 1:**
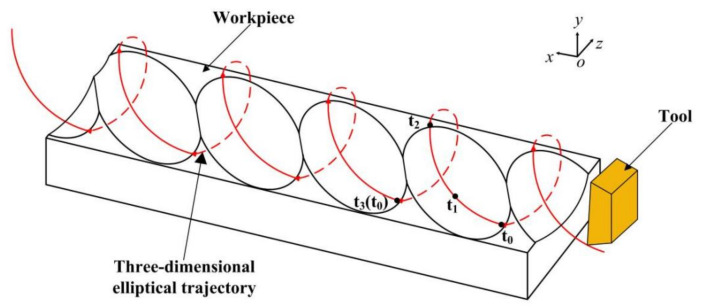
Principle of 3D-EVC.

The motion trajectory of the tool tip in 3D-EVC is shown in Figure 1. In each cutting cycle, the tool contacts the workpiece from point *t*_0_, from which the tool cuts the surface remaining from the previous cycle until point *t*_1_, which is the lowest point of the trajectory in the y direction, where the tool velocity is zero. The tool leaves the workpiece material at point *t*_2_, ending the effective cutting stage. The next cycle of cutting starts at point *t*_3_.

**Figure 2:**
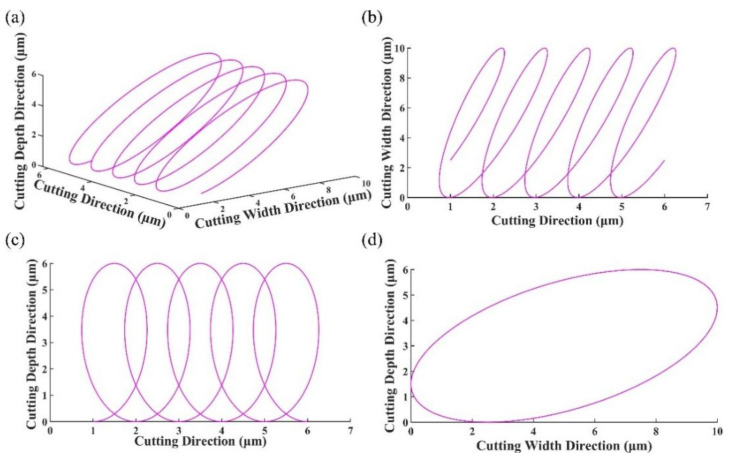
The trajectory and its projection of tool in 3D-EVC (**a**) 3D view (**b**) the projection of trajectory in XoZ plane (**c**) the projection of trajectory in XoY plane (**d**) the projection of trajectory in YoZ plane.

The trajectory projections of 3D-EVC on the cutting plane and vertical plane are both ellipses. The elliptical path of the tool is synthesized by the sinusoidal motion of three axes at a certain frequency. The motion equation of the tool can be summarized as follows:

(1) $$\left\{\begin{array}{l} x(t)=a\cos(\omega t+\varphi_{x})-v_{c}t\\ y(t)=b\cos(\omega t+\varphi_{y})\\ z(t)=c\cos(\omega t+\varphi_{z}) \end{array}\right.$$

where *a*, *b* and *c* are, respectively, the vibration amplitudes in *x*, *y* and *z* directions, *ω* is the vibration angular frequency. *φ_x_*, *φ_y_* and *φ_z_* are the initial phase angles in the *x*, *y* and *z* directions. *v_c_* is the cutting speed. As shown in Figure 2, by synthesizing the trajectory equations of the three axes, the spatial trajectory of 3D-EVC can be obtained. In addition, from the projection of each direction in Figure 2, it can be seen that 3D-EVC is reciprocating on the three projection planes.

Generally, the cutting speed of the tool is required to be lower than the maximum vibration speed during the 3D-EVC process to ensure the tool has separated from the workpiece and chip during each vibration cycle.

## 3. Finite Element Method

### 3.1. Material Constitutive Model

In the process of metal cutting, in order to ensure that the elastic-plastic deformation of the material in the simulation is the same as that in the real processing, the stress-strain relationship of the material must be determined reasonably in the simulation of metal cutting. In this paper, the Johnson-Cook constitutive model is adopted to describe the stress-strain relationship of metal materials. The Johnson-Cook constitutive model can be expressed as:

(2) $$\sigma =\left[A+B\varepsilon^{n}\right][1+c\ln(\frac{\dot{\varepsilon }}{\dot{\varepsilon_{0}}})]\left[1-(\frac{T-T_{r}}{T_{m}-T_{r}}{)}^{m}\right]$$

where $\dot{\varepsilon_{0}}$ is the reference strain rate, *A* is the elastic limit of the material, *B* is the hardening modulus, *C* is the strain rate dependency coefficient, *n* is the strain hardening index of the material, *m* is the thermal softening index of the material. *T_m_* is the melting temperature of the workpiece and *T_r_* is the ambient temperature. In this paper, the parameters of Johnson-Cook constitutive model are shown in Table 1.

### 3.2. The Criterion of Material Failure

In the finite element model of cutting process, the critical value of plastic strain accumulation is usually taken as the criterion of element failure. The material damage model selected in this study is the Johnson-Cook damage model, which is based on the equivalent plastic strain of each element. The damage principle of each element can be determined by:

(3) $$D=\sum_{}^{}\frac{\Delta \bar{\varepsilon }}{\varepsilon_{D}}$$

where *D* is the fracture failure parameter, $\Delta \bar{\varepsilon }$ is the equivalent plastic strain increment, $\varepsilon_{D}$ is the failure strain. When the fracture failure parameter *D* exceeds 1, the element begins to sustain damage, but at this point the element does not fail completely. The fracture failure strain of the material is expressed as follows:

(4) $$\varepsilon_{D}=\left[d_{1}+d_{2}\exp(d_{3}\frac{p}{q})\right]\left[1+d_{4}\ln(\frac{\dot{\varepsilon }}{\dot{\varepsilon_{0}}})\right]\left[1+d_{5}\left(\frac{T-T_{r}}{T_{m}-T_{r}}\right)\right]$$

where *p* and *q* are the compressive stress and von Mises stress, *d*_1_~*d*_5_ are the material failure constants, as shown in Table 2.

After the material is damaged, the simple stress-strain relationship cannot accurately express the damage failure behavior of the material. Hillerborg et al. [40] described the softening phenomenon of materials after damage by establishing the stress-displacement relationship, and defined fracture energy *G* to represent the energy required for cracking of materials per unit area, thus reducing dependence on the model mesh. In the damage evolution stage, the strain energy of the material is taken as the failure criterion, and fracture energy is the integral of the stress-strain of a material from the initial stage of damage to complete failure. It can be determined by:

(5) $$G_{f}=\int_{\bar{\varepsilon_{0}}}^{{\bar{\varepsilon }}_{f}}L_{0}\sigma_{y0}d\varepsilon =\int_{0}^{{\bar{u}}_{f}}\sigma_{y0}d\bar{u}$$

where ${\bar{\varepsilon }}_{0}$ and ${\bar{\varepsilon }}_{f}$ are the equivalent plastic strain at initial damage and material failure, *L*_0_ is the element feature length in the simulation model, *σ_y_*_0_ is the yield stress at initial damage, $\bar{u}$ is the equivalent plastic displacement of material failure process, ${\bar{u}}_{f}$ is the equivalent plastic displacement when the material fails completely.

In this paper, displacement-based failure is adopted as the material failure criterion. It can be seen from Equation (5) that the failure displacement value defined in ABAQUS is related to the element feature length *L*_0_ of the simulation model. Therefore, the initial failure displacement value is determined according to *L*_0_. Then, according to the comparison between the cutting force in the simulation and the cutting force in the experiment, the appropriate failure displacement value is finally obtained.

### 3.3. The Establishment of Finite Element Model

**Figure 3:**
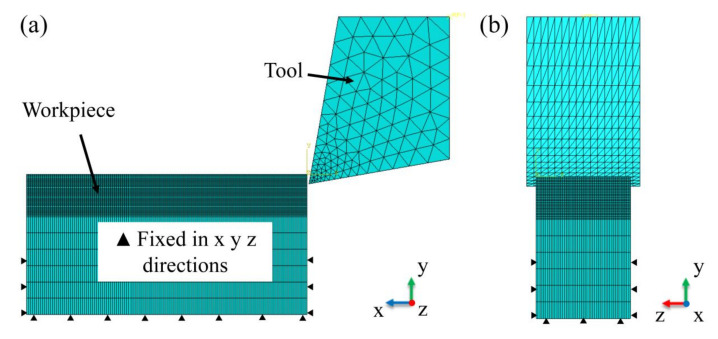
3D finite element cutting model (**a**) front view (**b**) left view.

The three-dimensional finite element model of EVC is shown in Figure 3. The model simulates the turning method in the actual process. Figure 3a is the front view of the model, and Figure 3b is the left view of the model. The model was established by ABAQUS software, and the workpiece size was 3 mm × 1.5 mm × 1 mm. Diamond tools are used in the model. The rake angle of the tool is 10 degrees, and the clearance angle of the tool is 10 degrees.

The material used for the workpiece is Ti6Al4V, and the material used for the tool is PCD. The material parameters of the tool and the workpiece are shown in Table 3. The tool material has high hardness, which is much higher than that of the workpiece material, so it is assumed that the tool is rigid.

The mesh type of the workpiece is set to be C3D8RT, and the number of meshes is 322,000. The mesh type of the tool is set to be C3D4T, and the number of meshes is 8037. In order to improve the calculation efficiency, the workpiece model is divided into two parts, namely chip layer and base layer. The chip layer is in contact with the tool to form chips. The base layer has imposed constraints to simulate the workpiece being clamped onto the machine tool. The mesh density of the chip layer is much higher than that of the base layer.

## 4. Results and Discussions

### 4.1. Effect of Cutting Parameters on Cutting Forces

**Figure 4:**
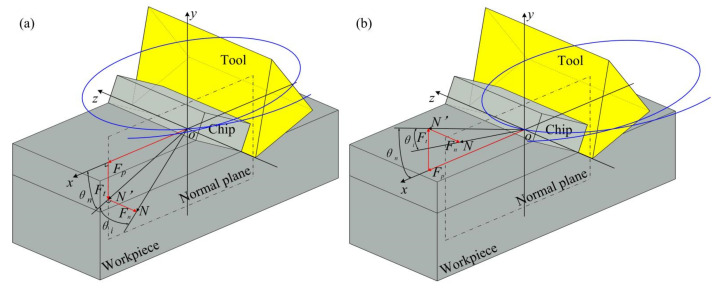
Analysis of the cutting force of the cutting tool acting on the workpiece (**a**) the stage similar to ordinary cutting (**b**) the stage of friction reversal.

The geometric relationship of each cutting force is shown in Figure 4, where *N* is the resultant force, *N*′ is the projection of the resultant force on the normal plane, *F_p_*, *F_t_* and *F_n_* are the principal force, thrust force and normal force respectively [41].

This section uses a single variable method to explore the effect of each cutting parameter on the cutting force. When exploring the effect of one cutting parameter on cutting force, the other cutting parameters take the default values. The default values are that the amplitude of the three directions of *x*, *y* and *z* is 15 μm, the vibration frequency is 20 kHz, the cutting depth is 0.1 mm, the cutting speed is 0.3 m/s and the rake and clearance angles of the tool are both 10 degrees.

#### 4.1.1. Effect of Cutting Speed on Cutting Force

**Figure 5:**
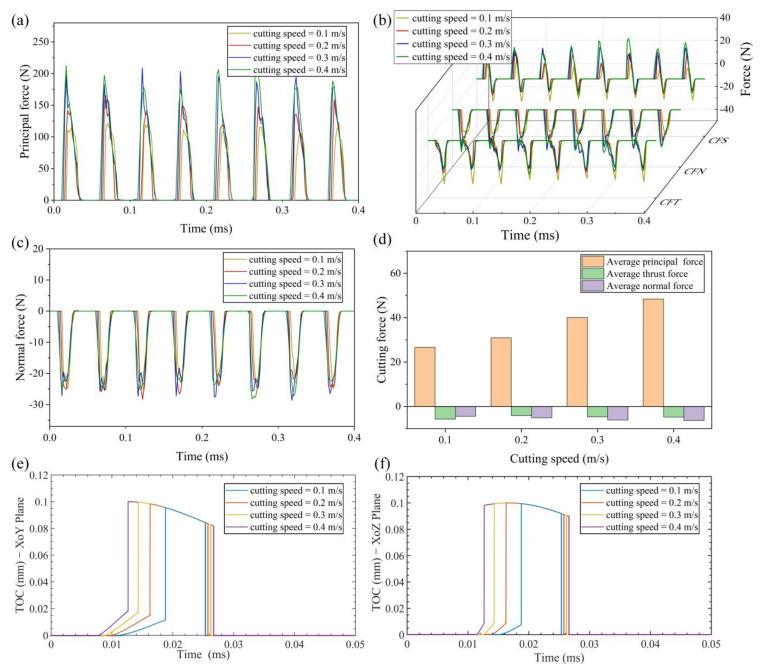
Results of cutting force and TOC with various cutting speeds (**a**) principal force (**b**) thrust force (**c**) normal force (**d**) average cutting force (**e**) TOC with various cutting speeds in XoY plane (**f**) TOC with various cutting speeds in XoZ plane.

Figure 5 shows the change of real-time cutting force and average cutting force at different cutting speeds. In addition, the curve of transient thickness of cut (TOC) changing with time is attached. It can observe the change of material removal under different cutting parameters. The cutting speed is altered between 0.1 m/s and 0.4 m/s with an interval of 0.1. All the above cutting speeds meet the condition of separate EVC.

The maximum principal force increases with the increase of cutting speed, and the average principal cutting force increases with the increase of cutting speed, as shown in Figure 5a,d. The average principal force is 26.62 N at 0.1 m/s, and 48.41 N at 0.4 m/s, increasing by 81.9%. The reason is that, as the cutting speed increases, the elliptical path of the cutting tool becomes longer over a cutting cycle. It means that there is a longer contact time between the tool and the workpiece. As shown in Figure 5e, after the tool has finished cutting the residual workpiece of the previous cycle, the TOC has an instantaneous increase. This trend intensifies with the increase of cutting speed. Moreover, more material is removed in a cutting cycle as the cutting speed increases. These result in an increasing of principal force peak value.

The thrust force consists of the force due to contact pressure and the force due to frictional stress. In this paper, CFT is the thrust force, CFN is the force due to contact pressure and CFS is the force due to frictional stress. It can be seen from Figure 5b that, with an increase of cutting speed, CFS increases gradually in the stage similar to ordinary cutting and decreases gradually in the stage of friction reversal. CFN increases gradually with increasing cutting speed. CFT is the combination of CFS and CFN. The effect of cutting speed on thrust force is lower when compared with principal force. This is also validated by the change trend of average thrust force.

As shown in Figure 5c, the maximum real-time normal force has little change with the change of cutting speed. This is because the maximum TOC at the various cutting speeds differ little as shown in Figure 5f. Thus, there is no significant difference in the maximum normal force at different speeds. In addition, increasing the cutting speed can increase the effective cutting time. The average normal force increases as the cutting speed increases. The average normal force is −4.38 N at 0.1 m/s, and −6.33 N at 0.4 m/s, increasing by 44.5%.

#### 4.1.2. Effect of Vibration Amplitude on Cutting Force

**Figure 6:**
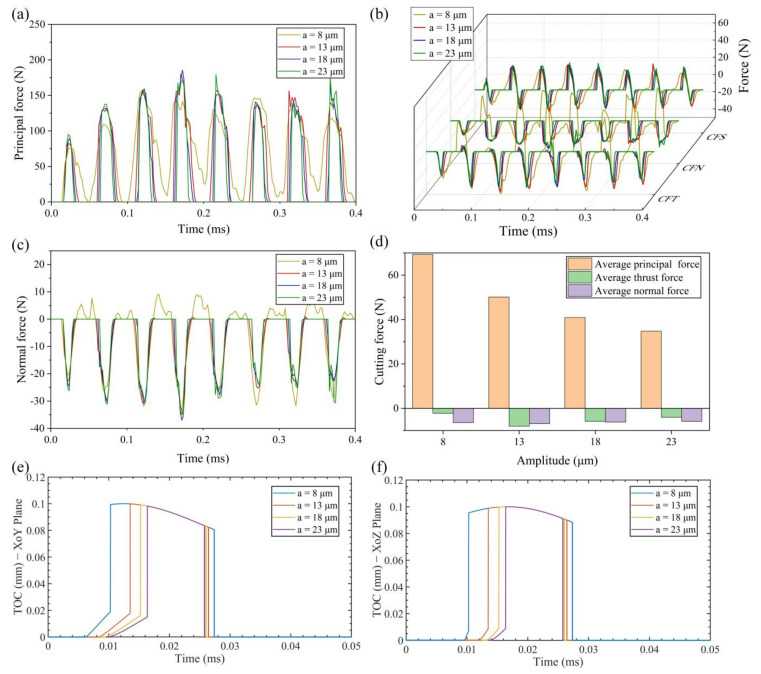
Results of cutting force and TOC with various amplitudes *a* (**a**) principal force (**b**) thrust force (**c**) normal force (**d**) average cutting force (**e**) TOC with various amplitudes *a* in XoY plane (**f**) TOC with various amplitudes *a* in XoZ plane.

**Figure 7:**
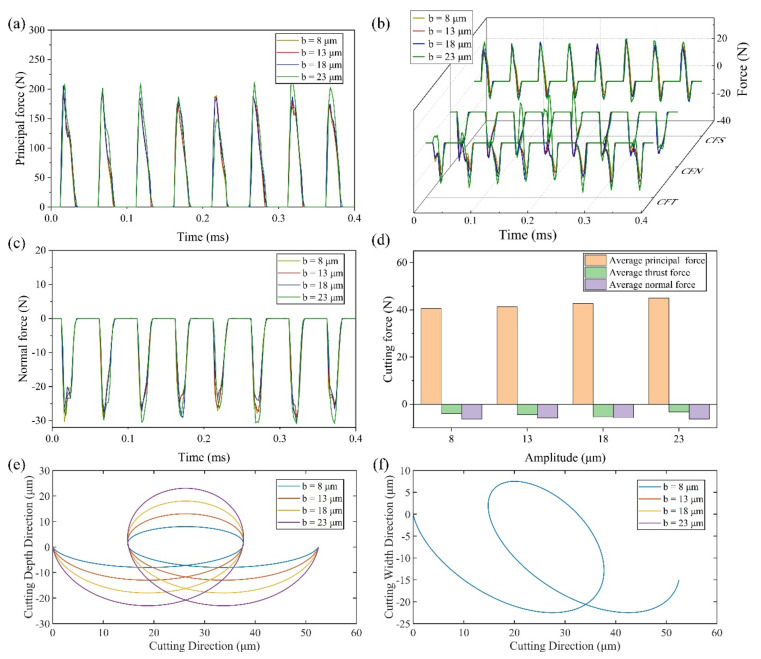
Results of cutting force and tool trajectories with various amplitudes *b* (**a**) principal force (**b**) thrust force (**c**) normal force (**d**) average cutting force (**e**) cutting trajectory with various amplitudes *b* in XoY plane (**f**) cutting trajectory with various amplitudes *b* in XoZ plane.

**Figure 8:**
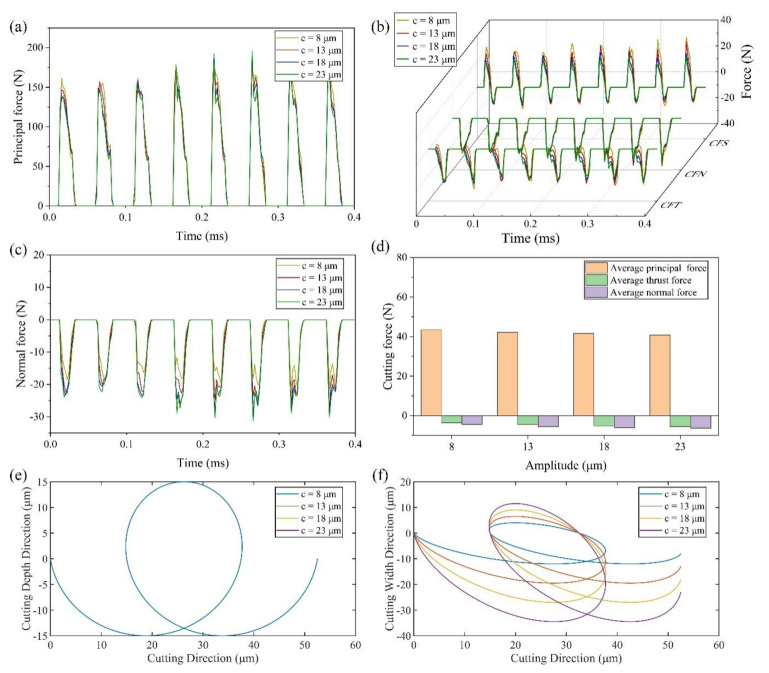
Results of cutting force and tool trajectories with various amplitudes *c* (**a**) principal force (**b**) thrust force (**c**) normal force (**d**) average cutting force (**e**) cutting trajectory with various amplitudes *c* in XoY plane (**f**) cutting trajectory with various amplitudes *c* in XoZ plane.

Figure 6, Figure 7 and Figure 8 show the change of real-time cutting force and average cutting force at different vibration amplitudes in three directions. The value of amplitudes *a*, *b* and *c* are set to 8 μm, 13 μm, 18 μm and 23 μm, respectively.

(1) Effect of amplitude *a*

As shown in Figure 6a, the maximum principal force increases gradually with the increase of amplitude *a*. The reason is that, after the tool finishes cutting the residual workpiece of the previous cycle, the TOC has an instantaneous increase. This trend intensifies with the increase of amplitude *a*, which makes the peak value of the principal force increase. On the other hand, the decrease of average principal force is the result of the decrease in speed ratio (*V_c_*/2*πfa*). With the increase of amplitude *a*, the effective cutting time decreases in a cutting cycle. It can be seen from Figure 6d that the average principal force is 69.32 N at 8 μm, and 34.75 N at 23 μm, decreasing by 49.9%.

As can be seen from Figure 6b, the CFS is almost unchanged in the stage similar to ordinary cutting, and gradually decreases in the stage of friction reversal with the increase of amplitude *a*. The CFN is almost constant with the increase of amplitude *a*. However, when the amplitude *a* is 8 μm, the tool and workpiece cannot be separated completely, which causes a slight change both in CFN and normal force. CFT is influenced by both CFN and CFS, and it decreases with the increase of amplitude *a*. It can also be seen from Figure 6e that the maximum TOC in XoY plane decreases with the increase of amplitude *a*. It means that, at the same cutting depth, the higher magnitude of amplitude *a*, the smaller the cutting resistance in the cutting depth direction. In addition, the average thrust force will decrease with the increase of amplitude *a* at the condition of separated EVC. It should be noted that the CFN in the stage similar to ordinary cutting is too large due to the continuous contact of tool and workpiece when the amplitude *a* is 8 μm, so the average thrust force is small. The average thrust force is −8.03 N at 13 μm, and −4.04 N at 23 μm, decreasing by 49.7%.

It can be seen from Figure 6f that the maximum TOC in XoZ plane in the cutting process remains unchanged with the increase of amplitude *a*. It makes the maximum normal force almost constant with the increase of amplitude *a*, as shown in Figure 6c. However, increasing amplitude *a* will reduce the effective cutting time within a single cutting cycle. Therefore, the average normal force decreases as the amplitude *a* increases at the condition of separated EVC. The average normal force is −6.87 N at 13 μm, and −5.87 N at 23 μm, decreasing by 14.6%.

(2) Effect of amplitude *b*

As demonstrated in Figure 7a,c, the real-time principal cutting force and the real-time normal force increase slightly with the increase of the amplitude *b*. The average principal force and the average normal force have the same trend with slightly changing shown in Figure 7d. The average principal cutting force and the average normal force are respectively 40.6 N and −6.21 N at 8 μm, 44.98 N and −6.27 N at 23 μm, increasing by 10.8% and 1%. Therefore, the changing of amplitude *b* has little effect on the principal force and normal force. The reason can be attributed to the same cutting trajectory in the XoZ plane shown in Figure 7f.

It can be seen from Figure 7b that, with the increase of amplitude *b*, CFS has little change in the stage similar to ordinary cutting, while increases gradually in the stage of friction reversal. CFN changes little except the singular period. The maximum thrust force increases gradually with the increase of amplitude *b* due to the increasing volume of material that needs to be removed, which can be deduced through the trajectories shown in Figure 7e. Additionally, the friction reversal effect was shown clearly since the positive thrust force appeared in the stage similar to ordinary cutting when the amplitude *b* is 23 μm. Therefore, a smaller average thrust force was obtained when the amplitude b is 23 μm.

(3) Effect of amplitude *c*

Figure 8a,b shows that the real-time principal cutting force and thrust force are slightly affected by the amplitude *c*. The reason is that the amplitude *c* mainly affects the inclination-degree of the tool elliptical trajectory. It has no significant effect on the cutting force in the cutting direction and the cutting depth direction since the cutting trajectory in XoY plane are the same as shown in Figure 8e. The average principal force shown in Figure 8d has the value of 43.43 N at 8 μm, and 40.74 N at 23 μm, decreasing by 6.2%.

With the increase of amplitude *c*, CFS decreases gradually in the stage similar to ordinary cutting, and nearly remains constant in the friction reversal stage. CFN is almost unchanged. Therefore, the average thrust force increases gradually with the increasing of amplitude *c*. The average thrust force is −3.65 N at 8 μm, and −5.52 N at 23 μm, increasing by 51.2%.

Figure 8c,d show the increase in the real-time normal force and average normal force when increasing the amplitude *c*. The average normal force is −4.43 N at 8 μm, and −6.35 N at 23 μm, increasing by 43.3%. It can be seen from Figure 8f that the higher the amplitude *c* is, the smaller the inclination of the elliptical trajectory of the tool is. The decrease of inclination of elliptical trajectory will lead to the increase of the displacement of the tool in the feed direction, which means that more material needs to be removed in the feed direction, i.e., the maximum normal force increases.

#### 4.1.3. Effect of Vibration Frequency on Cutting Force

**Figure 9:**
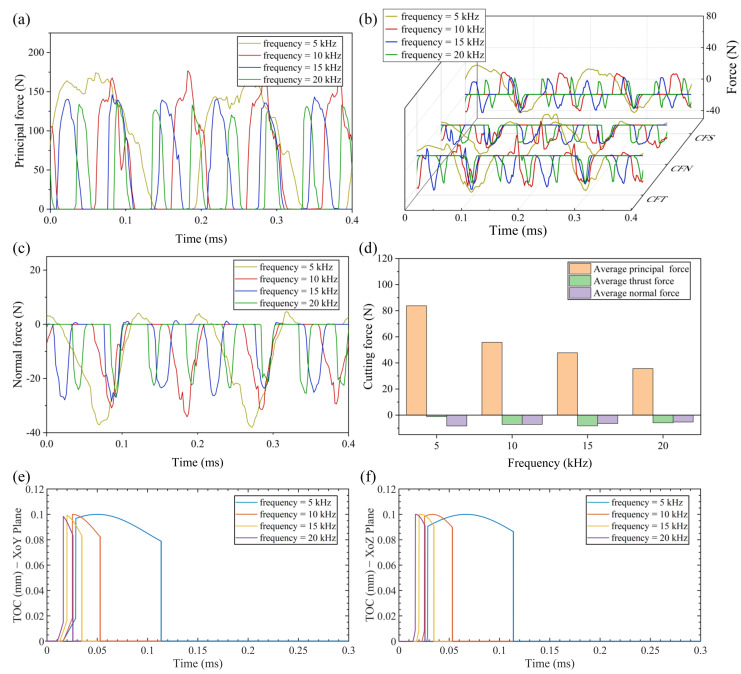
Results of cutting force and TOC with various vibration frequencies (**a**) principal force (**b**) thrust force (**c**) normal force (**d**) average cutting force (**e**) TOC with various vibration frequencies in XoY plane (**f**) TOC with various vibration frequencies in XoZ plane.

Figure 9a–c shows the changes of real-time cutting force with different vibration frequencies (5–20 kHz) at cutting speed of 0.2 m/s. It can be seen from Figure 9a–c that the real-time cutting forces in three directions decrease gradually with the increase of vibration frequency. As demonstrated in Figure 9d, the average principal cutting force and normal force decrease with the increase of vibration frequency. The decrease ratio from frequency of 5 kHz to frequency of 20 kHz are respectively 57.4% and 36.3%. However, the changing trend of average thrust force is increase first and then decrease due to the friction reversal effect. The critical cutting speed (2*πfa*) increases directly as the vibration frequency increases, which results in more cutting cycles at the same distance, as shown in Figure 9e,f. With the increasing of vibration frequency, the less volume of material needs to be removed in a single cutting cycle, resulting in a lower cutting component force in three directions.

#### 4.1.4. Effect of Cutting Depth on Cutting Force

**Figure 10:**
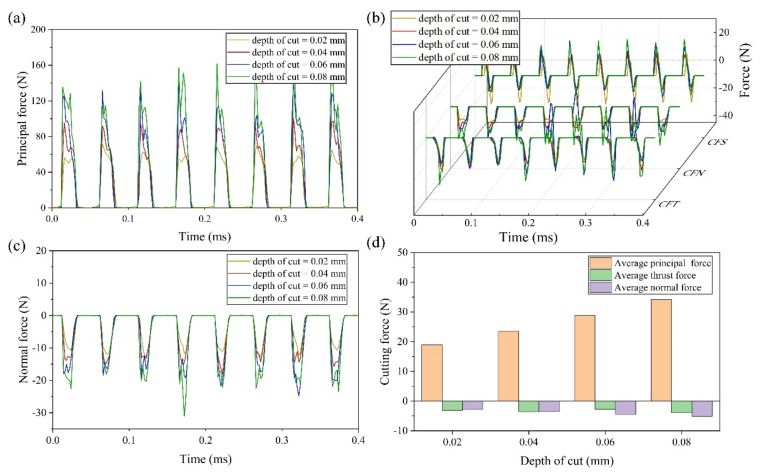
Results of cutting force with various cutting depths (**a**) principal force (**b**) thrust force (**c**) normal force (**d**) average cutting force.

Figure 10a–c shows the changes of real-time cutting force at different cutting depths (0.02–0.08 mm). It can be seen from Figure 10a–c that, with the increase of cutting depth, the real-time principal force, the real-time thrust force and the real-time normal force increase significantly. Figure 10d shows the changing trend of average cutting force. The average cutting force in three directions increases with the increase of the cutting depth, except the average thrust force at cutting depth of 0.06 mm. The abnormal value of average thrust force is due to the abnormal periods of CFN. The average forces in three directions are: 18.92 N, −3.11 N and −8.72 N at 0.02 mm, 34.13 N, −3.91 N and −13.12 N at 0.08 mm, increasing by 80.4%, 25.7% and 50.5%, respectively. The reason can be attributed to the cutting area in EVC being proportional to the cutting depth [19]. As the cutting depth increases, the volume of material that needs to be removed increases significantly, resulting in an increase in cutting force.

### 4.2. Effect of Cutting Parameters on Residual Stress

**Figure 11:**
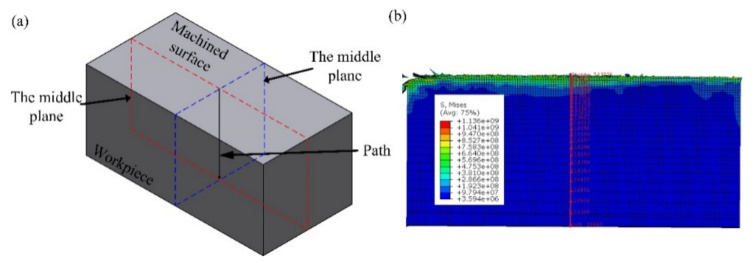
The path of obtained residual stress. (**a**) 3D view (**b**) front view.

Considering that residual stress analysis involves multiple analysis steps, in order to reduce the calculation time and improve the calculation accuracy, the model used in this section is modified: the workpiece size is 2 mm × 1 mm × 1 mm, and the total number of workpiece and tool meshes is 260,000 and 4506, respectively. The calculation process of residual stress in actual production is divided into four stages [42]: cutting, tool removal, boundary constraint removal and cooling. While the steps in the finite element simulation are set as cutting, tool removal and cooling. When cooling is set, the clamping mode of the workpiece is changed from fixing the bottom and both sides of the workpiece to fixing three endpoints of the workpiece, so that the boundary constraint removal step in actual production can be approximately simulated. As shown in Figure 11, the sampling path is set as the intersection line of two middle planes to study the effect of different cutting parameters on the residual stress.

This section also uses a single variable method to explore the effect of each cutting parameter on the residual stress. When exploring the effect of one cutting parameter on residual stress, the other cutting parameters take the default values. The default value is that the amplitudes of *a*, *b* and *c* are 10 μm, 15 μm and 18 μm, respectively, the vibration frequency is 20 kHz, the cutting depth is 0.08 mm, the cutting speed is 0.4 m/s and the rake and clearance angles of the tool are both 10 degrees.

#### 4.2.1. Effect of Cutting Speed on Residual Stress

**Figure 12:**
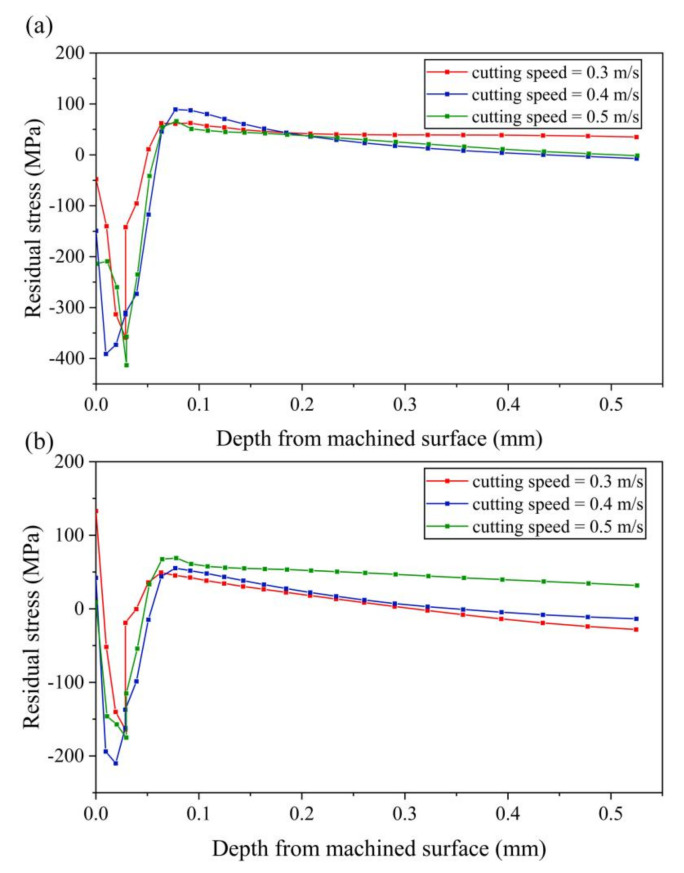
Results of residual stress with various cutting speeds (**a**) stress-xx (**b**) stress-zz.

Figure 12 shows the residual stress in three directions with different cutting speeds (0.3–0.5 m/s). Figure 12a shows that, when the cutting speed is 0.3 m/s, the stress-xx of the machined surface layer of the workpiece is compressive stress. As the distance from the machined surface increases, the compressive stress increases and reaches the maximum value when the distance from the machined surface is about 0.03 mm. Then the compressive stress gradually decreases and reversely changes to tensile stress. The tensile stress firstly increases to the maximum value and then decreases gradually and finally approaches zero. The variation trend of residual stress at 0.4 m/s and 0.5 m/s is similar to that at 0.3 m/s. However, with the increase of the speed, the compressive stress of the machined surface layer of the workpiece increases gradually, and the maximum compressive stress also increases gradually.

Figure 12b shows that the stress-zz of the machined surface layer of the workpiece is tensile stress. With the increase of cutting speed, the tensile stress of the machined surface layer of the workpiece decreases gradually. As the distance from the machined surface increases, stress-zz tends to decrease in tensile stress and changes to compressive stress. After the compressive stress reaches the maximum value, it decreases and then changes to tensile stress with the increases of the distance from the machined surface. Finally, tensile stress tends to a stable value close to zero.

#### 4.2.2. Effect of Vibration Amplitude on Residual Stress

**Figure 13:**
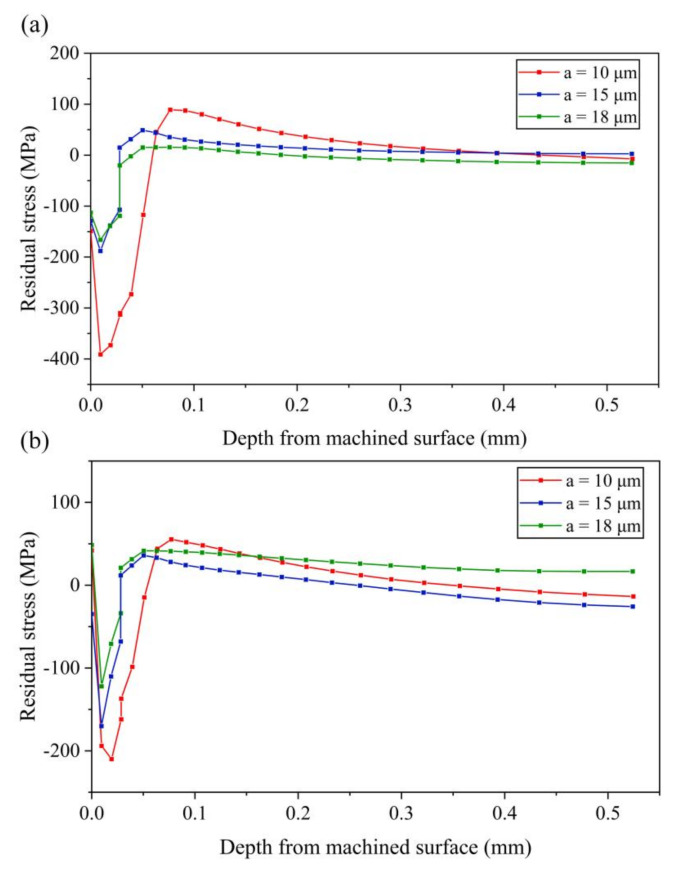
Results of residual stress with various amplitudes *a* (**a**) stress-xx (**b**) stress-zz.

**Figure 14:**
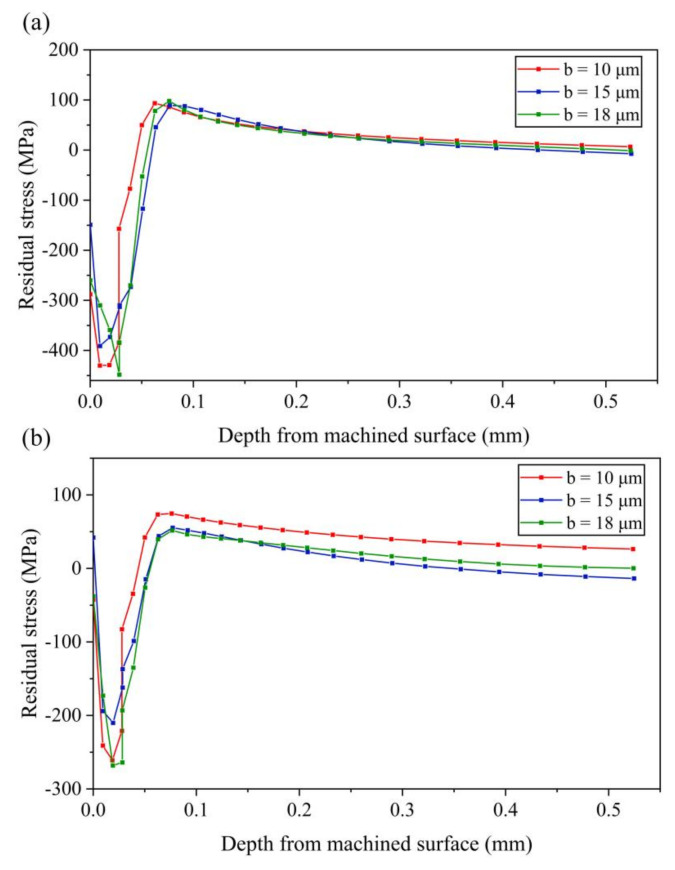
Results of residual stress with various amplitudes *b* (**a**) stress-xx (**b**) stress-zz.

**Figure 15:**
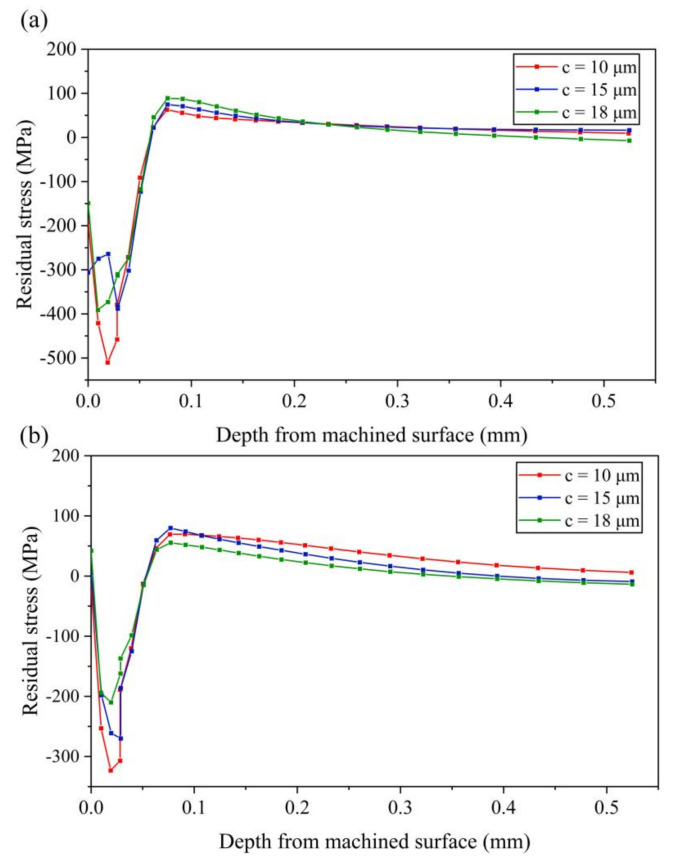
Results of residual stress with various amplitudes *c* (**a**) stress-xx (**b**) stress-zz.

Figure 13, Figure 14 and Figure 15 shows the change of residual stress at different vibration amplitudes in three directions, setting amplitudes *a*, *b* and *c*, to 10 μm, 15 μm and 18 μm, respectively.

(1) Effect of amplitude *a*

It can be seen from Figure 13a that the compressive stress of the machined surface layer of the workpiece decreases gradually with the increase of amplitude *a*. The maximum values of tensile stress and compressive stress decrease with the increase of amplitude. Especially when the amplitude *a* increases from 10 μm to 15 μm, both tensile stress and compressive stress decrease greatly. It can be concluded that, when the amplitude *a* is small, the stress-xx of the machined surface layer easily becomes a large compressive stress.

As shown in Figure 13b, with the increase of amplitude *a*, the maximum value of the compressive stress decreases, while the maximum value of tensile stress decreases first and then increases with the increase of amplitude *a*.

(2) Effect of amplitude *b*

It can be seen from Figure 14a that stress-xx changes little with the increase of amplitude *b*. The compressive stress on the machined surface layer of the workpiece decreases first and then increases with the increase of amplitude *b*. Both the maximum tensile stress and compressive stress decrease first and then increase with the increase of amplitude *b*. For stress-zz, the change trend of maximum compressive stress is the same as stress-xx, as shown in Figure 14b, while the maximum tensile stress decreases with the increase of amplitude *b*. According to the results of residual stress with vary amplitude *b*, it can be concluded that the effect of amplitude *b* on the residual stress is small.

(3) Effect of amplitude *c*

It can be seen from Figure 15a that, with the increase of amplitude *c*, the compressive stress on the machined surface layer increases first and then decreases. Increasing amplitude *c* can effectively reduce the maximum compressive stress. The maximum tensile stress increases with the increase of amplitude *c*, but the variation is small.

As can be seen from Figure 15b, the maximum compressive stress decreases with the increase of amplitude *c*. The maximum tensile stress increases first and then decreases. Therefore, it can be concluded that the large compressive stress can be obtained under the machined surface layer by setting a small amplitude *c*, which is helpful to improve the working performance of the workpiece.

#### 4.2.3. Effect of Vibration Frequency on Residual Stress

**Figure 16:**
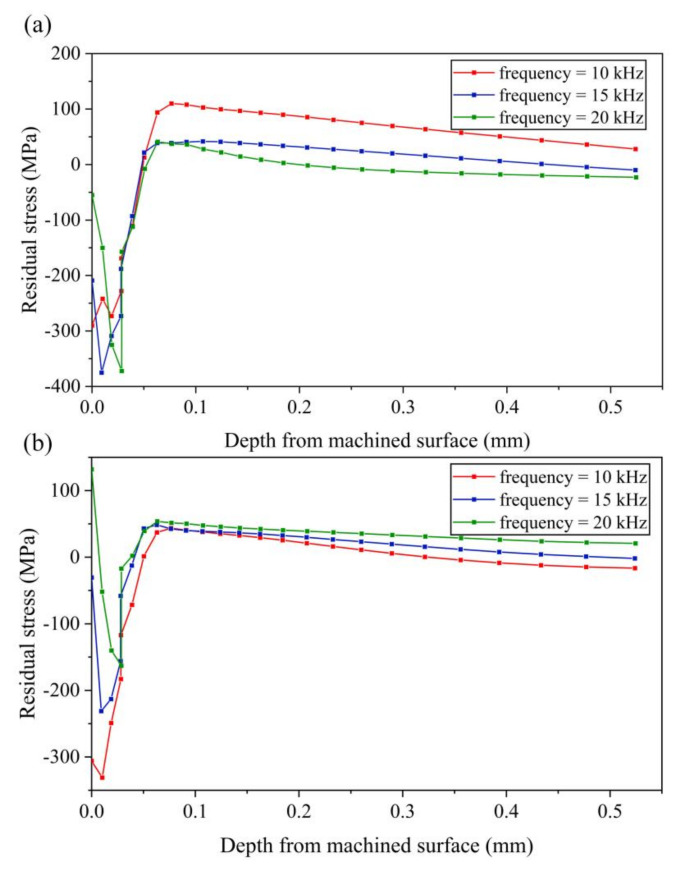
Results of residual stress with various vibration frequencies (**a**) stress-xx (**b**) stress-zz.

Setting different vibration frequencies to 10 kHz, 15 kHz and 20 kHz, respectively, the cutting speed is 0.3 m/s to ensure the cutting process be a separated EVC. The results of residual stress corresponding to different vibration frequency are shown in Figure 16.

As can be seen from Figure 16a, with the increase of vibration frequency, the stress-xx of the machined surface decreases gradually, and the maximum tensile stress also decreases gradually. As the distance from the machined surface increases, the tensile stress tends to zero with the increase of frequency. As shown in Figure 16b, with the increase of frequency, the maximum compressive stress of stress-zz decreases gradually, and the maximum tensile stress changes little.

#### 4.2.4. Effect of Cutting Depth on Residual Stress

**Figure 17:**
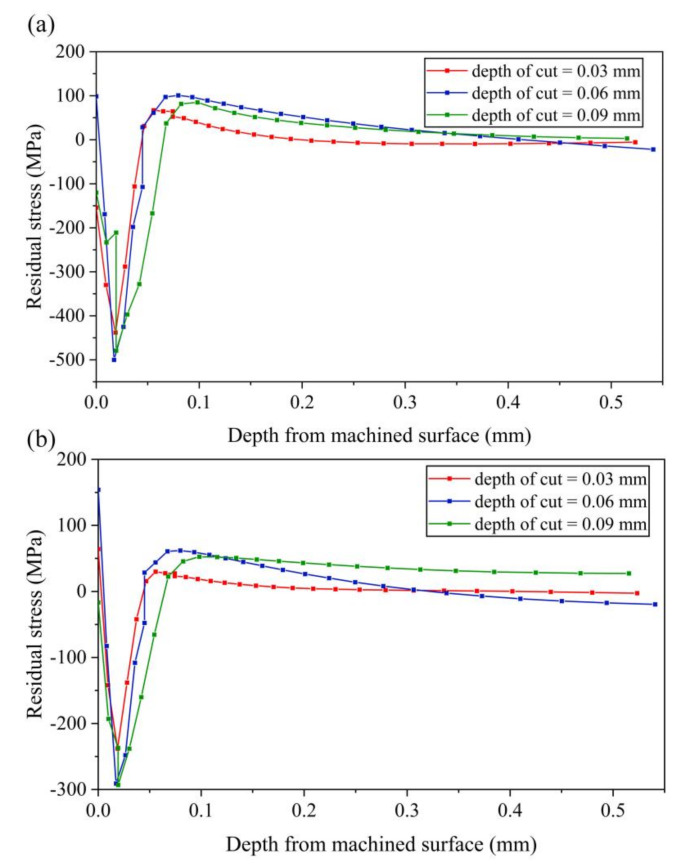
Results of residual stress with various cutting depths (**a**) stress-xx (**b**) stress-zz.

The effect of cutting depth on residual stress is shown in Figure 17. It can be seen that, with the increase of cutting depth, both the maximum tensile stress and the maximum compressive stress increase first and then decrease. The residual stress on machined workpiece surface also increases first and then decreases. Compared with the stress-xx at cutting depth of 0.08 mm shown in Figure 16a, it can be concluded that, with the increasing of cutting depth, it is much easier to obtain a larger magnitude of compressive stress layer near the machined surface. The same conclusion can be obtained for stress-zz when compared with the stress-zz at cutting depth of 0.08 mm shown in Figure 16b.

## 5. Conclusions

In this paper, the Finite Element Method was utilized to investigate the influence of processing parameters on cutting force and residual stress. A Johnson-Cook constitutive model was adopted to illustrate the plastic behavior of Ti6Al4V alloy. The kinematics of the 3D EVC were described first, and then the effect of vary cutting speed, vibration amplitude, vibration frequency and depth of cut on cutting force and residual stress were carried out and analyzed.

Compared with the effect of changing cutting parameters on thrust force and normal force, simulation results show that the change of cutting speed, vibration amplitude *a*, vibration frequency and depth of cut have larger effect on principal force. The principal force increases gradually with the increase of cutting speed from 0.1 m/s to 0.4 m/s. The increase ratio in average principal force can reach up to 81.9%. The maximum principal force increases with the increase of amplitude *a*. However, the average principal force decreases with the increase of amplitude *a* due to the decrease of effective cutting time in a cutting cycle. The decrease ratio is about 49.9% from amplitude of 8 μm to 23 μm. The principal force decreases with the increase of vibration frequency. The decrease ratio of average principal force is about 57.4% from a frequency of 5 kHz to 20 kHz. Meanwhile, the principal force increases with the increase of cutting depth. The decrease ratio of average principal force is about 80.4% from cutting depth of 0.02 mm to 0.08 mm. In addition, an interesting phenomenon should be noted: the friction reversal effect can not be found from the thrust force curve due to the combination of CFN and CFS, but this reversal effect does exist which can be seen clearly from the CFS curve.

Moreover, changing various cutting parameters have different effects on residual stress of machined workpiece surface in x direction and z direction. The simulation results show that the stress-xx on machined workpiece surface increases with the increase of cutting speed, decreases with the increase of amplitude *a* and vibration frequency. It first increases and then decreases with the increase of amplitude *c* and first decreases and then increases with the increase of amplitude *b* and cutting depth. The stress-zz on machined workpiece surface decreases with the increase of amplitude *b* and cutting speed. It first increases and then decreases with the increase of cutting depth. Meanwhile, it first decreases and then increases with the increase of amplitude *a*, amplitude *c* and vibration frequency. Above all, it can be seen from the study of residual stress that the compressive stress layer can be easily obtained near the machined surface by using 3D EVC, which is helpful to improve the working performance of workpiece.

## Figures and Tables

**Table 1:** Johnson-Cook parameters for Ti6Al4V alloy [38].

*A* (MPa)	*B* (MPa)	*C*	*n*	*m*	*T_m_* (°C)	*T_r_* (°C)
782.7	498.4	0.028	0.28	1	1660	20

**Table 2:** Johnson-Cook damage parameters [39].

*d*_1_	*d*_2_	*d*_3_	*d*_4_	*d*_5_	$\dot{\varepsilon_{0}}\;(s^{-1})$
−0.09	0.25	−0.5	0.014	3.87	0.001

**Table 3:** The material parameters of tool and workpiece [39].

Properties	Ti6Al4V	PCD
Density (kg/m^3^)	4440	14,450
Young’s modulus (GPa)	119	640
Poisson’s ratio	0.29	0.22
Specific heat (J/kg/K)	661	220
Thermal conductivity (W/m/K)	6.8	75.4

## Data Availability

Not applicable.
